# Abnormal gait, reduced locomotor activity and impaired motor coordination in *Dgcr2*-deficient mice

**DOI:** 10.1016/j.bbrep.2015.11.015

**Published:** 2015-11-18

**Authors:** Shin-ichiro Mugikura, Akira Katoh, Satoshi Watanabe, Minoru Kimura, Kagemasa Kajiwara

**Affiliations:** a Division of Basic Molecular Science and Molecular Medicine, Tokai University School of Medicine, Isehara, Kanagawa 259-1193, Japan; b Institute of Innovative Science and Technology, Tokai University, Hiratsuka, Kanagawa 259-1292, Japan; c Animal Genome Research Unit, National Institute of Agrobiological Sciences, Ikenodai, Tsukuba, Ibaraki 305-0901, Japan

**Keywords:** EGFP, enhanced green fluorescent protein, Dgcr2 gene, knock-out mice, 22q11.2 deletion syndrome, Motor coordination, Abnormal gait, Purkinje cell

## Abstract

It has been suggested that the *DGCR2* gene plays a role in the pathogenesis of 22q11.2 deletion syndrome. To analyze its function, we used our *Dgcr2*-knock-out/EGFP-knock-in mice (*Dgcr2*-KO mice). At 20-26 weeks of age, approximately 20% of *Dgcr2*-KO mice showed gait abnormalities with trembling and difficulty in balancing. Footprint test revealed awkward movements in *Dgcr2-*KO mice soon after they were placed on the floor. Once they started walking, their stride lengths were not different from wild-type mice. In short-term open field test, *Dgcr2*-KO mice travelled a significantly shorter distance and walked more slowly than wild-type mice during the initial 5 min after being placed in a new environment. In long-term open field test, *Dgcr2*-KO mice exhibited reduced cage activity compared to wild-type mice on the first day, but not on later days. *Dgcr2*-KO mice showed reduced latency to fall in the rotarod test, and the latency was not improved in the 3-day test. Histology revealed sparseness of cerebellar Purkinje cells in *Dgcr2*-KO mice. Our results suggest that *Dgcr2* plays a role in motor control related to Purkinje cell function and that the deficiency of *DGCR2* contributes at least to some of the symptoms of patients of 22q11.2 deletion syndrome.

## Highlights

• *Dgcr2*-KO mice showed abnormal behavior and gaits in footprint analysis.
• Locomotor activity was significantly reduced in *Dgcr2*-KO mice in open field tests.
• *Dgcr2*-KO mice showed impaired motor coordination in the rotarod test.
• *Dgcr2*-KO mice had sparseness of cerebellar Purkinje cells.
• Loss of *DGCR2* may contribute to neuronal dysfunction in humans.

## 1 Introduction

Previously, we isolated several cDNA clones whose expression was altered in murine primary cultures of embryonic neuron and brain upon stimulation with pentylenetetrazole, a seizure-inducing compound [1]. One of the downregulated genes, *Dgcr2* (*Sez12*) [2], is a mouse homolog of human *DGCR2* gene that is located in the human chromosome 22q11.2 region and encodes a C-type lectin-like transmembrane protein. It is known that heterologous microdeletion in chromosome 22q11.2 causes a syndrome termed 22q11.2 deletion syndrome (22q11.2 DS) [3]. 22q11.2 DS includes DiGeorge syndrome, conotruncal anomaly face syndrome, and velocardiofacial syndrome. Congenital heart disease and immune disorder are typically seen at birth in patients of 22q11.2 DS. Also common in 22q11.2 DS is growth retardation including delayed motor development [4], [5], facial dysmorphia, palatal anomalies, abnormal cerebellar morphology [6], [7], and early feeding problems [8], [9]. Other characteristic signs and symptoms of 22q11.2 DS include psychiatric disorders with abnormal behavior, autistic spectrum disorders [10], and unprovoked seizures [11], [12].

Individuals carrying the 22q11.2 microdeletion are also at risk of other psychiatric diseases, including anxiety and mood disorder [13], [14]. Recent evidence has indicated a relationship between the genes within the 22q11.2 region and the clinical features of 22q11.2 DS, such as schizophrenia [10], [13], [14], [15].

Our previous study in mutant mice suggested that the deletion of the gene for catechol-*O*-methyltransferase, rather than *Dgcr2*, is associated with an increased risk of schizophrenia in individuals of 22q11.2 DS [16]. However, it remains unclear which of the genes involved in the 22q11.2 microdeletion are responsible for other clinical features of 22q11.2 DS. Based on the previous findings that the human *DCGR2* is one of the genes involved in the microdeletion of 22q11.2 DS and the expression of *Dcgr2*, the mouse homolog of the human *DCGR2*, is affected by a seizure-provoking agent in cultured cells [2], we have hypothesized that the loss of *DGCR2* gene is involved in some of the neurological dysfunctions underlying the clinical manifestations of 22q11.2 DS, a product of complex genetic interactions.

To investigate the function of the *Dgcr2* gene products, we have recently generated *Dgcr2*-knock-out/EGFP-knock-in (*Dgcr2*-KO) mice by gene targeting [16]. The targeting vector carried a promotorless EGFP gene inserted into the upstream of the initiation codon of *Dgcr2* to visualize the pattern of *Dgcr2* expression.

In the present study, we examined the motor performance of *Dgcr2*-KO mice using footprint analysis, open field tests and rotarod test, and compared the results to those in wild-type mice to test our hypothesis that the symptoms of 22q11.2 DS patients involve the deficiency of *DGCR2* gene.

## 2 Materials and methods

### 2.1 Animals

Generation of *Dgcr2*-KO mice has been described [16]. Mice were crossed with C57BL/6 strain for at least 8 generations. Homozygous *Dgcr2*-KO and wild-type mice were obtained by mating heterozygous *Dgcr2*-KO mice and were identified by genotyping using PCR [16].

This study was in accordance with Guidelines for the Care and Use of Animals for Scientific Purposes at Tokai University and approved by the Institutional Review Board of Tokai University School of Medicine (Permit number: 151031).

### 2.2 Footprint analysis

The rear feet of mice were coated with non-toxic black ink, and each animal was allowed to walk on a 29.7 cm×42 cm absorbent paper. Stride lengths of the right and left rear feet were measured during the first 3–8 steps by using the footprints and movies.

### 2.3 Open field tests

Two types of open field test were conducted. For short-term open field test, each mouse was placed at a corner of a plastic 60 cm×60 cm×40 cm box, and the movement was recorded with a CCD camera from above for the initial 5 min. A 30 cm×30 cm square area at the center of the floor was designated as “the center zone”. A video behavior analysis software, Smart (Panlab Co., Barcelona, Spain), was used to measure horizontal travelled distance, the numbers of entries from the outer zone to the center zone and from the center zone to the outer zone, resting time and walking speed for each mouse in the first 5minutes after the animal was placed on the floor. Resting time was defined as the time when the mouse was moving slower than 4 cm/s. Walking speed was calculated after excluding resting time. For long-term open field test, each mouse was placed in a transparent acrylic 37 cm×24 cm×27 cm box, and cage activity and the cumulative rearing frequency were recorded with an infrared sensor (SUPERMEX, MUROMACHI Kikai Co., Tokyo, Japan) under a 12-h light/12-h dark condition. Mice were placed in the box during the light phase. The first light (3 h) and dark (12 h) phases, which generally are regarded as a period of acclimatization or searching, were designated as Day 0. Cage activity on Day 0 was expressed in counts per minute, and that on Day 1–3 were determined separately for the light and dark phases and expressed in an arbitrary unit. A water bottle and a food container were equipped in the box. The test was performed for four consecutive days for each animal.

### 2.4 Rotarod test

A rotarod treadmill (MUROMACHI Kikai Co., Tokyo, Japan) consisting of a gridded plastic rod (5 cm in diameter) was used. Mice were given one day to become acclimatized with the rotarod apparatus. On the testing day, a mouse was placed on the rod rotating at 5 rpm, and the latency to fall was measured. Each mouse was given three trials per day for a maximum of 300 s in each trial. The test was performed on three consecutive days. The test was performed at 10 rpm as well.

### 2.5 Hanging wire grip test

A standard wire cage lid was used. The perimeter of the cage lid was wrapped with aluminum foils to prevent the mouse from walking off the cage lid. Each mouse was placed on the wire cage lid, and the lid was turned upside down at a height of approximately 20 cm. We measured the time until the mouse fell from the cage lid. A 60-s cutoff time was used.

### 2.6 Blood calcium concentration

Mice at 12–16 weeks of age were euthanized by intraperitoneal administration of 74 mg/kg body weight pentobarbital, and blood was sampled via cardiac puncture. To measure blood calcium concentrations, Calcium E-HA test (Wako Pure Chemical Ltd., Osaka, Japan) was used. This method is based on the formation of a complex between serum calcium and methyl xylenol blue under alkaline conditions (pH 12).

### 2.7 Immunohistochemistry

Mice were deeply anesthetized and perfused with 3% heparin in phosphate buffered saline (PBS), followed by 4% paraformaldehyde. A sagittal half of the cerebellum was taken, fixed in 4% paraformaldehyde for 16 h at 4 °C and embedded in paraffin, and serial 5-µm sections were prepared. Prior to immunostaining, the sections were treated with 3% hydrogen peroxide and then with 10% normal goat serum in PBS for 1 h at room temperature. The sections were incubated with rabbit anti-GFP antibody (ab290, Abcam, Cambridge, UK; at 1:500 dilution) overnight at 4 °C. After washing with PBS, the sections were incubated with horseradish peroxidase-labeled goat anti-rabbit antibody (ab6721, Abcam; at 1:1000 dilution) for 60 minutes at room temperature. The expression of EGFP was visualized using 3,3,-diaminobenzidine tetrahydrochloride as chromogen. Sections of wild-type cerebellum were used as negative controls. The sections were photographed with Zeiss Imager A2 microscope (Zeiss, Germany). Frozen 10-µm sections of the cerebellum were also prepared and stained with rabbit anti-ß III tubulin antibody (D71G9, Cell Signaling Technology, Danvers, Massachusetts, USA; at 1:200 dilution) and Alexa Flour 594-labeled goat anti-rabbit antibody (ab150080, Abcam; at 1:500 dilution). Sections were photographed by confocal microscopy with Zeiss LSM 700 (Zeiss, Germany).

### 2.8 Statistical analysis

Values were expressed as the mean±SEM. Differences were tested by *t*-test, ANOVA or post-hoc Scheffe's test as appropriate. All statistical analyses were done by using IBM SPSS Statistics version 22 (IBM Japan Co., Tokyo, Japan). A *p*-value<0.05 was considered significant.

## 3 Results

### 3.1 Gait

**Fig. 1:**
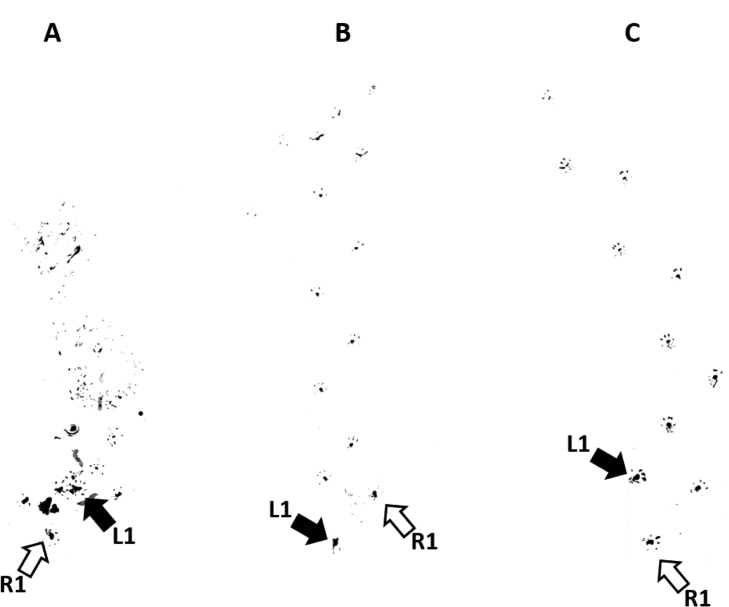
Footprint test. A representative of abnormal footprint patterns seen in *Dgcr2*-KO mice (A). As can be seen also in Supplementary movie 1, apparent gait abnormalities were observed after the mice started walking in some but not all *Dgcr2*-KO mice. A representative of normal footprint patters seen in other *Dgcr2*-KO mice (B) indistinguishable from that seen in wild-type mice (C). These mice were 22-24 weeks old and correspond to the mice in Supplementary movie 1–3. L1 and R1 indicate the starting point of the left foot and right rear foot, respectively.

At 20-26 weeks of age, some *Dgcr2*-KO mice exhibited apparent gait disturbance with slight trembling and difficulty in keeping balance while walking. Two of the ten representative *Dgcr2*-KO mice we examined showed awkward and uneasy movement with ill-balanced steps immediately after being placed on the floor for footprint analysis. Fig. 1A shows gait abnormality of *Dgcr2*-KO mice. This characteristic behavior lasted for a few tens of seconds and was repeatedly observed. Once *Dgcr2*-KO mice started walking, they walked more slowly than wild-type mice with trembling as shown in Supplementary movie 1. The remaining eight *Dgcr2*-KO mice showed normal gaits (Fig. 1B and Supplementary movie 2) similar to that of wild-type mice (Fig. 1C and Supplementary movie 3). We measured the mean stride lengths of the right and left feet in all ten *Dgcr2*-KO mice including the two exhibiting abnormal gaits and in eight wild-type mice. The mean stride length was not different between *Dgcr2*-KO and wild-type mice (Table 1).

**Table 1:** Undistinguishable findings between wild-type and *Dgcr2*-KO mice.

	WT	KO	*p*-Value
Stride length (mm)			
Left foot	62.5±2.3	53.4±4.7	0.13
Right foot	61.1±1.1	54.5±3.7	0.16
*n*	8	10	
			
Blood calcium			
concentration (mg/dl)	7.9±0.3	8.3±0.3	0.43
*n*	6	6	
			
Hanging wire			
Grip test (sec)	60.0±0.0	56.6±2.5	0.36
*n*	9	18	

Values are mean±SEM. Differences between the groups were tested by *t*-test. Abbreviations are: WT, wild-type mice; KO, *Dgcr2*-KO mice.

Supplementary material related to this article can be found online at 10.1016/j.bbrep.2015.11.015.

The following is the Supplementary material related to this article Movie 1, Movie 2, Movie 3.

Movie 1 We took movies when the mice were placed on the floor for footprint analysis. Supplementary movie 1shows the Dgcr2-KO mice on Fig. 1A.

Movie 2 Supplementary movie 2 shows the Dgcr2-KO mice on Fig. 1B.

Movie 3 Supplementary movie 3 shows the wild-type mouse on Fig. 1C.

### 3.2 Open field tests

**Fig. 2:**
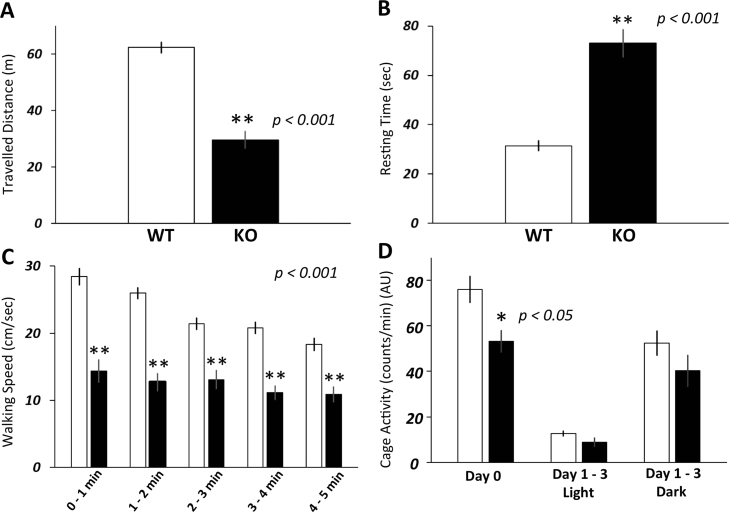
General locomotor activity in open field tests.Travelled distance (A), resting time (B) and walking speed (C) in the first five minutes after the animals were placed in the box for short-term open field test were compared between *Dgcr2*-KO (KO; filled bars) and wild-type mice (WT; open bars). *Dgcr2*-KO mice showed lower locomotor activity (** *p*<0.001 by *t*-test) and slower motion than wild-type mice (*p*<0.001 by ANOVA, ** *p*<0.001 by post-hoc Scheffe’s test). The numbers of analyzed animals were 10 and 15 for wild-type and *Dgcr2*-KO mice, respectively. Cage activity (D) in long-term open field test was compared between *Dgcr2*-KO and wild-type mice. Cage activities on Day 1 to 3 were in an arbitrary unit (AU) and averaged. The numbers of analyzed animals were 6 and 10 for wild-type and *Dgcr2*-KO mice, respectively. Cage activity on Day 0 was significantly lower in *Dgcr2-*KO mice than in wild-type mice (* *p*<0.05 by *t*-test). Error bars indicate SEM.

*Dgcr2*-KO mice showed abnormal gaits right after being placed on the floor, whereas their stride lengths were comparable to those of wild-type mice. To investigate in more detail about the locomotor activity of *Dgcr2*-KO mice, we employed two types of open field test. In short-term open field test, the travelled distance of *Dgcr2*-KO mice was significantly shorter than that of wild-type mice (*p*<0.001) (Fig. 2A). The numbers of entries from the outer zone to the center zone and from the center zone to the outer zone were also significantly reduced in *Dgcr2*-KO mice (10.0±2.0 and 9.8±1.9, respectively) compared to wild-type mice (15.4±1.2 and 15.4±1.2, respectively; *p*<0.05). Resting time was significantly longer in *Dgcr2*-KO than in wild-type mice (Fig. 2B). Walking speed was significantly slower in *Dgcr2*-KO mice than in wild-type mice through the entire five minutes of observation (Fig. 2C). Thus, locomotor activity was impaired in *Dgcr2*-KO mice not only for several tens of seconds after they were placed on the floor as seen in the footprint test but also for at least five minutes or more.

Next, long-term open field test was conducted to examine whether such locomotor activity impairments persist for a longer time. In long-term open field test, the cage activity on Day 0 was significantly reduced in *Dgcr2*-KO mice compared to that in wild-type mice (*p*<0.05; Fig. 2D). However, their cage activities on Day 1–3 were comparable for both the dark and the light phases (Fig. 2D). No significant difference was found in the rearing frequency between *Dgcr2*-KO and wild-type mice (Day 0, 3.0±0.4 in wild-type and 2.0±0.3 in KO mice; Day 1–3 light phase, 0.4±0.0 in wild-type and 0.4±0.2 in KO mice; Day 1–3 dark phase, 2.5±0.5 in wild-type and 1.9±0.8 in KO mice). These results demonstrated that the locomotor activity abnormalities of *Dgcr2*-KO mice were temporally limited to when they were placed in a new environment.

### 3.3 Rotarod test

**Fig. 3:**
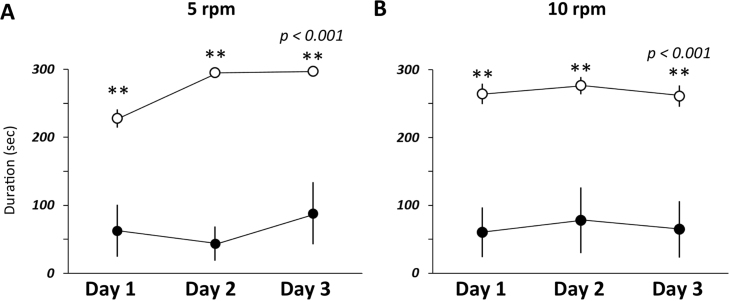
Motor coordination and motor learning in rotarod test. The latencies on the rod rotating at 5 rpm (A) and 10 rpm (B) were compared between *Dgcr2*-KO mice (filled circles) and wild-type mice (open circles). Each trial was performed with the cutoff period of 300 s. *Dgcr2-KO* mice showed a shorter latency than wild-type mice (*n* = 8 per group; *p*<0.001 by ANOVA; ** *p*<0.001 by post-hoc Scheffe's test). Error bars indicate SEM.

To examine if *Dgcr2*-KO mice have abnormalities not only in locomotor activity but also in forced motor activity or motor learning, we used the rotarod test. Our results showed that the latency to fall was significantly shorter in *Dgcr2*-KO mice than in wild-type mice at both 5 and 10 rpm (*p*<0.001; Fig. 3). In addition, the latency at 5 rpm became longer in wild-type mice during the 3-day test, whereas no significant improvement was found in *Dgcr2*-KO mice (Fig. 3).

### 3.4 Hanging wire grip test and Blood calcium concentration

Since 22q11.2 DS patients manifest tremor caused by neuromuscular abnormality and/or hypocalcemia [17], [18], [19], we conducted the hanging wire grip test and measured blood calcium concentrations in *Dgcr2*-KO and wild-type mice. No significant differences were found in the hanging wire grip test or blood calcium concentrations between *Dgcr2*-KO and wild-type mice (Table 1).

### 3.5 Morphology of Cerebellar Purkinje cells

**Fig. 4:**
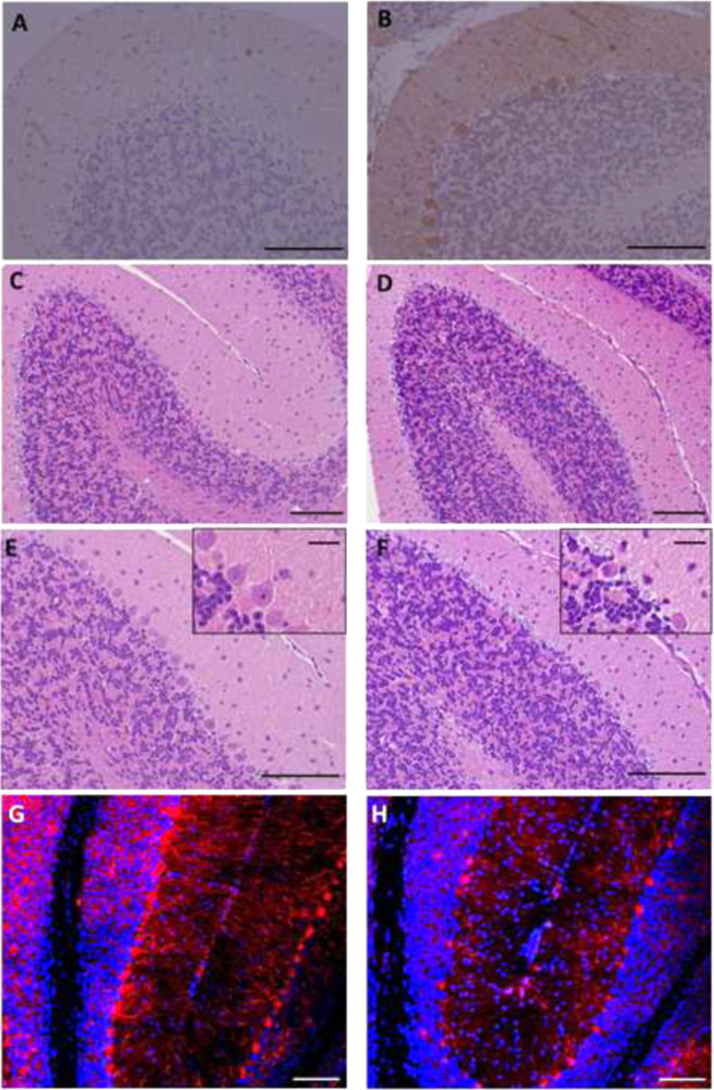
Morphology of Purkinje cells. EGFP immunostaining of the cerebellum from wild-type (A) and *Dgcr2*-KO mice (B). Knocked-in EGFP was strongly expressed in Purkinje cells of *Dgcr2*-KO mice. Hematoxylin and eosin staining of the cerebellum from wild-type (C and E) and *Dgcr2*-KO mice (D and F). Immunostaining of the cerebellum from wild-type (G) and *Dgcr2*-KO mice (H) using anti βIII tubulin antibody. The number of Purkinje cells appeared reduced in *Dgcr2*-KO mice compared to that in wild-type mice. The scale bars represent 20 μm in the insets and 100 μm in others.

The expression of the knocked-in EGFP was intense in cerebellar Purkinje cells and relatively weak in the cerebellar molecular layer in *Dgcr2*-KO mice (Fig. 4A and B), suggesting that *Dgcr2* is strongly expressed in Purkinje cells. Hematoxylin and eosin staining showed sparseness of cerebellar Purkinje cells in *Dgcr2-*KO mice (Fig. 4D and F) compared to the distribution of Purkinje cells in wild-type mice (Fig. 4C and E). Anti-ß III tubulin antibody staining also showed Purkinje cell reduction in *Dgcr2*-KO mice (Fig. 4G and H). These results suggest that *Dgcr2* contributes to the establishment of Purkinje cells.

## 4 Discussion

In this study, we demonstrated that *Dgcr2*-KO mice exhibited reduced general locomotor activity in the open field tests and poor motor coordination in the rotarod test. We previously demonstrated ubiquitous expression of *Dgcr2* including muscle cells in mice [2]. Our present results further suggested that *Dgcr2* expression in the brain, particularly the expression in cerebellar Purkinje cells, plays a role in regulating locomotor activity and coordination. This notion arises because a loss of Purkinje cells was observed in *Dgcr2*-KO mice whereas no abnormalities were found in the morphology of muscle cells (data not shown) or in the strength of the muscles (the hanging wire grip test; Table 1).

Cerebellar hypoplasia was found in some but not all patients with a 22q11.2 deletion [7]. In addition, it was reported that young patients with a 22q11.2 deletion including *DGCR2* gene showed significant motor deficits [4]. In our study, *Dgcr2*-KO mice exhibited deficits in locomotor activity and motor coordination, suggesting that motor deficits observed in patients of 22q11.2 DS are caused by the loss of *DGCR2* gene, while *Dgcr2*-KO mice had no gross morphological abnormalities such as hypoplasia in the brain. Such a difference is probably caused by the fact that the portion of the genome deleted in 22q11.2 DS contains multiple genes, which usually results in severer phenotypes than a single gene deletion.

We demonstrated that the EGFP knocked-in under the promotor of *Dgcr2* was strongly expressed in Purkinje cells (Fig. 4B). It is likely that a C-type lectin-like transmembrane protein, the product of *Dgcr2* gene, is essential for establishing or maintaining functional Purkinje cells. A previous study revealed disrupted migration of parvalbumin-positive interneurons in the cortex in LgDel mouse, a model of 22q11.2 DS having a loss of *Dgcr2* gene locus [20]. Although a partial loss of Purkinje cells was found and the existing Purkinje cells appeared morphologically normal in *Dgcr2*-KO mice (Fig. 4), it is possible that those Purkinje cells did not function normally. Further studies are required to elucidate how *Dgcr2* contributes to the establishment and maintenance of functional Purkinje cells.

In footprint analysis, approximately 20% of *Dgcr2*-KO mice showed gait abnormality and the remaining 80% of KO mice showed normal gaits (Fig. 1 and Supplementary movies 1,2,3). Yet, general locomotor activity and motor coordination were significantly impaired in all of *Dgcr2*-KO mice. In addition, reduced locomotor activity appeared mainly after *Dgcr2*-KO mice were placed in a new environment, whereas their cage activity increased over time, reaching a level comparable to that in wild-type mice in three days (Fig. 2D). These findings can explain why our recent study found the behavior of *Dgcr2*-KO mice indistinguishable from that of wild-type mice [16]. To date, a loss of Purkinje cells has been reported in a number of spontaneous mutant mice and gene-manipulated mice [21]. Even when a large number of Purkinje cells were lost, as seen in *lurcher* and *pcd* mice, a diversity of impairments in motor coordination and ataxic behavior were observed [22], [23], [24]. The low penetration of the effects of a loss of *Dgcr2* gene and the temporally-limited impairment of locomotor activity observed in *Dgcr2*-KO mice might reflect the partial loss of Purkinje cells resulting in mosaic dysfunction of the cerebellum or regional specificity of *Dgcr2* deficiency.

22q11.2 DS is known as a hemizygous deletion syndrome [3]. Patients with this syndrome manifest not only motor deficits but idiopathic seizure, attention deficit, and anxiety [5], [6], [15]. Such characteristic symptoms associated with 22q11.2 DS may require functional defects of other genes within 22q11.2 than *DGCR2*. However, we cannot exclude the possibility that *DGCR2* deficiency is related to emotional and social abnormalities frequently observed in patients of this syndrome because abnormal locomotor activity of *Dgcr2*-KO mice manifested itself only when the mice were placed in a new environment and, thus, is temporally limited.

In summary, *Dgcr2*-KO mice showed temporally-limited deficits in locomotor activity, impaired motor coordination and motor learning, and sparseness of cerebellar Purkinje cells. Our results provide a clue to therapeutic strategies for diseases caused by complex genetic interactions, such as 22q11.2 DS. Utilizing combination of multiple animal models will help understand a number of human diseases.
